# Examining the effect of advertising film-supported activities in Turkish courses on the critical thinking skills of seventh grade students

**DOI:** 10.3389/fpsyg.2026.1873339

**Published:** 2026-08-18

**Authors:** Ayşe Şimşek, Nurullah Aydın, Sıddık Bakir

**Affiliations:** 1 Turkish Language Teacher, Turkiye Cumhuriyeti Milli Egitim Bakanligi, Ankara, Türkiye; 2 Department of Turkish and Social Sciences Education, Kazım Karabekir Faculty of Education, Atatürk Üniversitesi, Erzurum, Türkiye; 3 Department of Turkish and Social Sciences Education, Kazım Karabekir Faculty of Education, Kazım Karabekir Eğitim Fakültesi, Erzurum, Türkiye

**Keywords:** advertising films, critical thinking skills, mixed methods research, student, Turkish language education

## Abstract

Although advertising films have increasingly been used as authentic multimedia resources in language education, empirical evidence on their effectiveness in fostering students’ critical thinking skills remains limited. Moreover, the mechanisms through which advertising films support higher-order thinking remain underexplored from constructivist, multimodal learning, and cognitive engagement perspectives. This study examined the effect of advertising film-based activities on the critical thinking skills of seventh-grade students in Turkish language classes using an explanatory sequential mixed-methods design. Quantitative data were collected through a quasi-experimental pretest-posttest control-group design, followed by classroom observations and semi-structured interviews to explain the findings. The study involved 44 seventh-grade students over a twelve-week intervention. The experimental group participated in advertising film-based learning activities, whereas the control group followed the standard curriculum. Quantitative results showed that the experimental group significantly outperformed the control group in critical thinking skills (*p* < 0.05), indicating a strong intervention effect, while no significant difference was found between post-test and retention-test scores, suggesting sustained learning gains over time. Qualitative findings indicated increased student engagement, active participation, and deeper cognitive engagement, which supported and contextualized the quantitative findings. These findings suggest that advertising films enhance critical thinking through cognitive engagement, multimodal meaning-making, and active knowledge construction grounded in constructivist learning theory. Beyond Turkish language education, the study contributes to international research on multimedia-supported learning by demonstrating how authentic audiovisual materials can foster higher-order thinking skills in diverse educational contexts.

## 1 Introduction

Critical thinking is widely recognized as one of the essential competencies for individuals living in increasingly complex, information-rich societies (Facione, 1990). Beyond acquiring knowledge, students are expected to interpret information critically, evaluate competing perspectives, identify implicit meanings, and construct well-reasoned judgments (Halpern, 2014). Consequently, fostering critical thinking has become a central objective of language education, where learners are expected not only to comprehend texts but also to question, interpret, and reconstruct meaning through active engagement with language (Freire and Macedo, 1987; Luke, 2012). However, despite this educational goal, conventional language instruction continues to rely predominantly on printed texts and teacher-centred practices that often emphasize comprehension and recall rather than higher-order cognitive processes (Darling-Hammond, 2020). As a result, students may have limited opportunities to develop the analytical, interpretive, and evaluative skills required for critical thinking (Brookhart, 2010).

In response to these challenges, multimedia-supported instructional approaches have gained increasing attention because they provide authentic learning environments in which learners actively construct meaning through interaction with multiple forms of representation (Mayer, 2014; Moreno and Mayer, 2007). From a constructivist perspective, learning is enhanced when students engage with authentic materials that encourage inquiry, discussion, and reflection rather than passively receiving information (Bruner, 1966; Vygotsky, 1978). Likewise, multimodal learning emphasizes that meaning is created through the interaction of visual, verbal, auditory, symbolic, and narrative resources, enabling learners to process information more deeply and from multiple perspectives (Jewitt, 2009; Kress, 2010). These theoretical perspectives suggest that multimodal materials can stimulate cognitive engagement and support the development of higher-order thinking skills by encouraging learners to compare, interpret, evaluate, and reconstruct meaning (Cope and Kalantzis, 2009).

Among multimodal materials, advertising films constitute particularly rich learning resources because they combine visual, verbal, symbolic, emotional, and narrative elements within a short and highly condensed format (Dyer, 1982). Rather than simply conveying commercial messages, advertising films present authentic social and cultural narratives that invite viewers to identify persuasive strategies, interpret implicit meanings, evaluate ideological positions, and reflect on alternative viewpoints (Machin, 2007; O’Halloran, 2004). These characteristics make advertising films potentially valuable instructional tools for promoting critical thinking in language education. Nevertheless, although previous research has demonstrated the educational value of multimodal texts, short films, and advertising materials (Mills, 2010; Serafini, 2014), empirical evidence examining advertising films as multimodal literary texts through classroom-based experimental interventions remains limited. In particular, studies investigating their effects on both the development and retention of middle school students’ critical thinking skills are scarce.

Addressing this gap, the present study investigates the effects of advertising film-based activities on the critical thinking skills of seventh-grade students in Turkish language classes using an explanatory sequential mixed-methods design. By integrating constructivist learning, multimodal learning, and critical literacy perspectives, the study aims not only to examine the effectiveness of advertising films as instructional materials but also to provide a theoretical explanation of how multimodal literary texts can foster higher-order thinking through cognitive engagement, critical interpretation, and reflective meaning-making. In doing so, the study contributes to the growing international literature on multimedia-supported language learning, multimodal literacy, and critical thinking development.

### 1.1 The literary potential of advertising films in language education

The contemporary definition of literature extends beyond traditional written texts to include oral, visual, and digital narratives. Luukka (2021) views literature as a social structure shaped by multimodal texts across time and space, while Calafato and Hunstadbråten (2024) highlight its broad scope, encompassing short stories, comics, audiobooks, and digital narratives. These perspectives suggest that literature is a dynamic form of meaning-making in which multiple semiotic resources contribute to interpretation. Consequently, literature serves not only as a means of developing linguistic competence but also as a context for promoting higher-order thinking and critical literacy.

Complex literary texts encourage learners to analyse multiple perspectives, interpret symbolic meanings, and critically evaluate different viewpoints (Calafato and Hunstadbråten, 2024; Tsang et al., 2023). Similarly, studies have shown that engagement with literary texts enhances analytical thinking (Aydın and Yıldız, 2024; Bobkina et al., 2021), while literature-based instructional practices strengthen students’ critical reading skills (Calafato, 2024). From a constructivist and multimodal learning perspective, these processes are supported when learners actively construct meaning by integrating visual, verbal, and symbolic information.

Advertising films, which combine visual, auditory, verbal, and narrative elements within authentic communicative contexts, represent a distinctive form of multimodal literary text. Their layered meanings, persuasive discourse, and symbolic structures encourage learners to question implicit messages, compare alternative perspectives, and reflect on how meaning is constructed. Accordingly, integrating advertising films into language classrooms provides both a theoretical and practical foundation for fostering critical literacy and developing students’ critical thinking skills.

### 1.2 Advertising films as a medium for enhancing critical thinking in language education

In today’s educational context, critical thinking is essential for students to make informed decisions in complex information environments. In language education focused on meaning-making and communication, analysis and evaluation skills are central. Integrating these skills into instruction deepens students’ engagement with texts and strengthens their ability to question and infer. Demirbağ et al. (2016) found that even without direct instruction, a supportive classroom climate can increase tendencies toward critical thinking. Advertising films, with their intense, multi-layered content delivered in a short time, can effectively support these cognitive processes. Stanley and Lawson (2020) demonstrated that analyzing advertising texts enables students to recognize persuasion strategies and engage in inference-based thinking. Advertising films thus bridge the gap between media literacy and critical thinking. Their linguistic and rhetorical features, including repetition, metaphors, and rhetorical questions, offer rich material for analysis (Koonnala and Chaiwong, 2023). This helps students question the functional aspects of language. Similarly, Sharmin (2023) emphasized that creating multimodal narratives enhances critical evaluation skills. Zalani et al. (2024) also demonstrated that short, layered content can foster critical thinking more effectively than traditional methods. Advertising films offer a comprehensive learning environment that fosters these skills through both symbolic and verbal cues.

### 1.3 Teaching Turkish with a focus on critical thinking and multimodal literacy

The 2023 Turkish Language Teaching Program, implemented by the Ministry of National Education (2023), aims to holistically develop students’ thinking, comprehension, and expression skills from 5th to 8th grade. Beyond teaching basic language skills, the program supports higher-order abilities such as critical thinking and working with multimodal texts. To foster deeper thinking, many learning outcomes include elements that promote it. Skills such as making inferences, recognizing different viewpoints, identifying the author’s perspective, and proposing alternative solutions are systematically addressed at each grade level. This helps students move beyond simple comprehension to questioning, discussing, and reconstructing texts. Speaking and writing activities are designed to encourage empathy, consider opposing views, and build coherent arguments.

Responding to the demands of the digital age, the program emphasizes the effective use of multimodal texts. It aims to develop students’ ability to create meaning across media by combining written texts with graphs, tables, images, and multimedia such as cartoons, comics, videos, and online content. In analyzing these texts, students are encouraged to critically examine the accuracy, intent, and impact of messages, thereby supporting digital and visual literacy. The assessment process aligns with these goals: alongside knowledge, higher-order thinking skills, such as analysis, interpretation, and problem-solving, are also evaluated. This approach ensures that students are assessed not only on what they know but also on how they use and interpret information, fostering multidimensional learning and contributing to their academic and intellectual growth.

### 1.4 Literature review

Previous studies suggest that multimodal literary texts can support the development of critical thinking by encouraging learners to analyse, interpret, and evaluate meaning through multiple semiotic modes. For example, Checa-Romero (2016) reported that video games and short films enhanced students’ comparative thinking and meaning-making across contexts, whereas Farías and Seguel (2014) showed that advertising texts promoted the analysis of ideological structures. Likewise, Gainer (2012), Gomes and Machado (2020), and Huang (2017) demonstrated that multimodal text production and analysis foster democratic awareness, media criticism, and critical interpretation. Similarly, Öman (2017), Öman and Hashemi (2015), Pantaleo (2021), and Stewart et al. (2025) emphasized that multimodal texts strengthen students’ awareness of visual language, symbolic meaning, and the integration of visual and verbal elements.

Although these studies provide evidence that multimodal materials contribute to higher-order thinking, they primarily focus on media production, text analysis, or general multimodal literacy (Checa-Romero, 2016; Farías and Seguel, 2014; Gainer, 2012; Gomes and Machado, 2020; Huang, 2017; Öman, 2017; Öman and Hashemi, 2015; Pantaleo, 2021; Stewart et al., 2025). Empirical research examining advertising films as multimodal literary texts implemented through classroom-based instructional interventions remains limited. Furthermore, a Web of Science Core Collection search conducted in June 2024 using combinations of the keywords advertising film, commercial, multimodal text, and critical thinking identified no experimental studies investigating the effects of advertising film-based instruction on middle school students’ critical thinking skills or the retention of these skills. Consequently, empirical evidence regarding the effectiveness of advertising film-based instructional interventions in this context remains limited.

### 1.5 Research questions

The purpose of this study is to examine the effect of advertising-supported film activities on the critical thinking skills of 7th-grade students in Turkish language lessons. In this study, critical thinking skills were assessed using the Critical Thinking Skills Scale. The scale evaluates students’ critical thinking dispositions across five dimensions: curiosity and systematicity, open-mindedness, analyticity, self-confidence, and truth-seeking. These dimensions reflect students’ tendencies to approach texts and information critically by questioning evidence, considering different perspectives, analysing information systematically, making independent judgments, and seeking accurate and justified conclusions. Higher scores indicate higher levels of critical thinking skills. Accordingly, the quantitative research questions focus on changes in students’ critical thinking skills scale scores and the retention of these skills following the instructional intervention. The study seeks to answer the following research questions:

1. Does the implementation of advertising film-supported activities significantly increase the critical thinking skills scores and retention levels of students in the experimental group?
2. Are there any significant differences in critical thinking scores between students in the experimental group who participated in advertising film-supported activities and those in the control group who learned through textbook-based activities?
3. What observations were made by researchers during the event?
4. What are the opinions of the experimental group students regarding the activity process?

## 2 Method

This study employed an explanatory sequential mixed-methods design. The quantitative phase was conducted first to examine the effects of advertising film-based activities on seventh-grade students’ critical thinking skills. Research Questions 1 and 2 were addressed through a quasi-experimental pretest–posttest control-group design by comparing students’ critical thinking scores and retention levels before and after the intervention.

Following the quantitative phase, qualitative data were collected through classroom observations and semi-structured interviews with students in the experimental group to provide a deeper understanding of the quantitative findings. Specifically, Research Question 3 was addressed through researchers’ classroom observations, while Research Question 4 was explored through students’ opinions regarding the implementation process. In line with the explanatory sequential mixed-methods design, the qualitative findings were used to explain and elaborate on the quantitative results by providing contextual insights into how advertising film-based activities contributed to the development of students’ critical thinking dispositions, particularly curiosity and systematicity, open-mindedness, analyticity, self-confidence, and truth-seeking, as assessed by the Critical Thinking Skills Scale.

### 2.1 Quantitative dimension of the research

In the quantitative dimension of the research, a pretest-posttest control-group quasi-experimental design was used. In this design, groups are determined by the researcher, but assignments are not random. At the beginning of the experimental process, the researcher administers a pre-test to both the experimental and control groups. While the experimental procedure is applied to the experimental group, students in the control group continue their education within the standard teaching program. At the end of the process, the final test is administered to both groups, and the data obtained are analyzed and evaluated using statistical methods (Creswell, 2014).

### 2.2 Qualitative dimension of the research

In the qualitative dimension of the research, semi-structured interviews and classroom observations were used to support and explain the quantitative findings.

#### 2.2.1 Student interviews

A semi-structured interview form was used to support the quantitative findings obtained from the implementation. Semi-structured interviews were conducted with students in the experimental group to obtain their opinions regarding the advertising film-supported activities.

#### 2.2.2 Observation

Classroom observations were conducted to support the quantitative findings and to complement the interview data. To ensure objectivity, multiple observers were involved in the observation process. The researcher initially observed the experimental group’s activities as a complete observer. In addition, three teachers at the school where the research was conducted were selected as observers using criterion sampling. A faculty member from the Department of Turkish Language Education at Atatürk University served as a participant observer in a supportive (secondary) role, while a Turkish-language teacher at the research site assumed the role of a full participant observer.

### 2.3 Participants

#### 2.3.1 Determining the working group for the quantitative phase

Quantitative data were collected using purposeful sampling, a nonprobability sampling method. In this regard, individuals with specific qualities related to the research topic were selected to form the study group. In the first stage, meetings were held with officials from the National Education Directorate in the district where the research was to be conducted, and the previous year’s High School Transition Exam (LGS) school success rankings were obtained. Based on these data, a school with an average level of success was identified. The selected school was evaluated within the scope of the research because it has the potential to reflect the city’s socio-economic structure, and it was deemed suitable following interviews with school administrators. Subsequently, two classes with a balanced gender distribution and similar class sizes were selected to form the experimental and control groups, following the recommendation of Fraenkel et al. (2012) that comparison groups in quasi-experimental studies should be as similar as possible in their initial characteristics. The study was conducted with 44 students, divided into two classes of 22 each.

The selection and identification of participants were carefully conducted in accordance with the research’s objectives and scope. Participating students came from families with similar socioeconomic conditions, and this homogeneity enables the findings to be interpreted within the context of students attending schools with comparable socioeconomic characteristics. Regarding gender distribution, the groups were formed in a balanced manner, with 21 male and 23 female students. There were no participants with special needs. Additionally, most students have been attending the same educational institution since fifth grade and have demonstrated similar academic achievement levels. This similarity in participants’ educational backgrounds and academic performance contributes to a more reliable interpretation of the data.

#### 2.3.2 Identification of participants for the qualitative stage

In the qualitative phase of the research, participants were selected from the experimental group using stratified sampling. Based on their Turkish language course grades from the previous academic year, students were classified into three achievement levels: low, medium, and high. Students from each achievement level were included in the interviews to ensure variation in academic achievement.

Semi-structured interviews were conducted with 22 students from the experimental group. The students were organized into four small interview groups consisting of six, six, five, and five students, respectively. The interviews were conducted to obtain students’ opinions regarding the advertising film-supported activities and their experiences during the implementation process.

#### 2.3.3 Ethics approval and consent to participate

Before data collection began, ethical approval was obtained from the Atatürk University Social and Human Sciences Ethics Committee, Educational Sciences Unit Ethics Committee (Approval ID: 01, Date: December 30, 2024). The study was conducted in accordance with the ethical principles outlined in the Declaration of Helsinki.

Following ethical approval, the study site was identified as a public school located in the Eastern Anatolia Region of Türkiye, and the necessary institutional permissions were obtained from the school administration.

Written informed consent forms were collected from the parents of participating students on December 15, 2024. Prior to the consent process, students and their legal guardians were informed about the purpose, scope, procedures, and voluntary nature of the study. Participation was confirmed through signed consent forms obtained before the implementation of the intervention.

### 2.4 Data collection tools

During data collection, the Critical Thinking Skills Scale (CTSS) was administered, followed by observation and interview forms.

#### 2.4.1 Observation form

Students in the experimental group were systematically observed to analyze their behaviors related to the learning process in detail. To conduct this process effectively, three researchers served as full observers. The inclusion of multiple researchers in the participant observation process has enabled the collection of richer, more multidimensional data, allowing for a comprehensive presentation of different aspects of the phenomenon under study through the various perspectives of the researchers (Hammersley and Atkinson, 1995).

The researchers received a total of 4 hours of observation training on different days over a two-week period. The training program outlines the purpose of observation, the behaviors to be observed, the evaluation criteria, and the data recording methods in detail. Before proceeding to the actual observation, a preliminary application was carried out with twelve students representing the sample group. This preliminary study helped identify potential problems and difficulties that may arise in applying the observation form, enabling the necessary adjustments to be made before the form was incorporated into the actual research process. To ensure the pilot program is carried out effectively, a school with similar conditions has been carefully selected. The observation form covers various dimensions, including the physical and social learning environment, the process of entering the lesson (attracting attention, arousing curiosity, and reminding students of prior knowledge), the implementation of activities, forms of classroom interaction (student-teacher and student–student interactions), and assessment processes. During the observation period, students’ cognitive skills, such as analyzing, interpreting, evaluating, discussing, and generating original ideas, were observed. In this regard, the impact of the experimental application on students’ critical thinking skills has been revealed.

#### 2.4.2 Semi-structured interview form

In the study, semi-structured interviews were conducted to elicit students’ opinions in the experimental group about the application. The semi-structured interview technique is considered a more suitable research method in the field of education because it provides a certain level of standardization while allowing the researcher flexibility (Türnüklü, 2000). In developing interview questions, relevant literature was reviewed, program outcomes were examined in detail, and questions were prepared based on the data collected. Additionally, student feedback and expert opinions played a significant role in shaping this project’s development. The interview form was reviewed by a measurement and evaluation expert and three Turkish teachers. Following expert advice, the last two questions in the form were reordered, and the two indicated words were replaced with synonymous expressions. After completing the necessary arrangements, trial interviews were conducted with four students who were not included in the application to assess the clarity of the questions. Based on the findings, it was determined that the questions were generally understandable, and the interview form was finalized and made ready for implementation.

### 2.5 Implementation process

The application process lasted 12 weeks. Advertising films for the experimental group were carefully selected to align with curriculum outcomes and students’ age levels. Expert opinions informed the selection, emphasizing values education, imagination, critical thinking, empathy, and social awareness. Of 36 advertising films pre-evaluated on YouTube, 12 were selected for their educational suitability. The Ministry of National Education’s Regulation on Textbooks and Educational Tools was considered during selection. The selected films were integrated into lesson plans designed to stimulate imagination, analytical thinking, and creativity.

The selection criteria included visual narrative elements (Mayer, 2014), language and discourse (Gee, 2012), thematic coherence (Kress and Van Leeuwen, 2020), subject matter (Sherin, 2003), the educational function of characters (Bandura, 2009), age appropriateness (Tomlinson, 2005), message clarity (Chang, 2022), creativity (Mishra and Koehler, 2006), and audience impact (Green and Brock, 2000). The advertising films were used solely for educational purposes and had no commercial ties. The 12 selected films and their weekly schedule are presented in Table 1.

**Table 1:** Advertising texts determined for the test group.

Week	Advertising text
1	TEMA Vakfı-“Ağaç Kalsın” Çevre Bilinci Reklamı
2	Ziraat Bankası-Sünnet Temalı Geleneksel Değerler Reklamı
3	Cola Turka-Farkındalıklar Üzerine Mizahi Toplumsal Reklam
4	Ziraat Bankası-153. Yıl Kurumsal Kimlik ve Değerler Reklamı
5	Kent-Bayramda Paylaşma ve Aile Temalı Reklam Filmi
6	Türk Hava Yolları- Hayaller ve Seyahat Temalı Yolculuk Reklamı
7	AB İnsan Hakları-Kısa Film Yarışması Birincilik Ödüllü Film
8	Pegasus- Gençlik ve Hayallerinin Peşinden Gitme Temalı Reklam
9	Türk Hava Yolları- Hayal Kurma ve Gerçekleştirme Temalı Reklam
10	Karagöz ve Hacivat-Gölge Oyunu ile Eğitimsel Tanıtım Filmi
11	Türk Hava Yolları-Geleceğe Dönüşüm “Sürdürülebilirlik Reklamı
12	Ekopet-Geri Dönüşüm ve Çevre Bilinci Temalı Reklam Filmi

The implementation process took 14 weeks. The first week was spent on process planning and preparatory meetings, while the final week was devoted to completing the implementation and conducting overall evaluations. During the remaining 12 weeks, activities were based on the specified advertising films. During the application process, students in the experimental group participated in structured activities for 2 hours each week to develop their skills in understanding, analyzing, and critically evaluating films. Tasks such as watching, discussing, and interpreting texts were designed to support the acquisition of the learning outcomes included in the Turkish language teaching program. The activities were implemented using various teaching techniques, including question-and-answer sessions, role-playing, small-group discussions, brainstorming, and mind mapping. Every week, these methods were used to carry out processes related to the film. On the other hand, activities included in the textbook were implemented in the control group and were structured to develop basic and critical thinking skills. The students in the control group studied the textbook material and completed the workbook exercises. These applications have helped students consolidate the knowledge they have acquired. The activities in both groups were conducted by classroom teachers using different techniques and methods. The approach adopted by teachers emphasizes creating learning environments that foster student participation and interaction.

### 2.6 Data analysis

For quantitative data, the data were first defined, and missing data were imputed. Subsequently, the normality of the data and the homogeneity of variance were checked; it was concluded that the pre-test and post-test data showed normal distributions. For quantitative data analysis, dependent- and independent-samples t-tests were used, and descriptive statistics were computed. The t-test can be used to determine whether the means of two groups are different. When both an experimental and a control group are used, this is an appropriate method for testing the effectiveness of the experiment applied to the experimental group (Field, 2013).

#### 2.6.1 Observation data analysis

To analyze the observational data, the data were first classified, then coded, categorized, and thematized. The data were organized around themes such as environmental information, lesson introduction, activity phase, classroom interaction, and assessment activities. These themes helped categorize the data meaningfully and ensured a systematic analysis process. Thematic analysis and interpretation were then conducted, focusing on student interactions, participation, and assessment skills within each theme. Student behaviors and responses were systematically evaluated across stages, including lesson introduction, activity participation, interaction dynamics, and assessment practices. Meaningful findings were obtained regarding the development of students’ critical thinking skills, including creativity, problem-solving, evaluation, and interpretation. Reliability and validity assessments supported the robustness of the analysis.

#### 2.6.2 Analysis of interview forms

Data from the interview forms were coded and analyzed. Thematic analysis was conducted through a systematic coding process in which initial codes were generated from the data and subsequently organized into broader themes. Similar expressions were grouped under the same code tag. Two independent coders conducted this process to enhance reliability and validity, and the inter-rater reliability coefficient was calculated. The coefficient is expected to be above 70% (Miles and Huberman, 1994). This value was calculated as 87%. Commonly identified codes were listed and grouped into categories. Categories were structured to represent broader themes. Thus, the data from the interview forms were organized into a meaningful, systematic structure (Creswell, 2014). To ensure the credibility and dependability of the qualitative findings, peer debriefing and audit trail procedures were employed throughout the analysis process. In addition, researcher reflexivity was maintained by continuously reflecting on potential biases during data interpretation. It should be noted that the qualitative findings are context-specific and therefore not intended for statistical generalization, but rather for analytical transferability to similar educational contexts.

### 2.7 Reliability and validity

A series of reliability and validity measures was implemented throughout the research. The study employed a mixed-methods approach, with multiple researchers reviewing and verifying data during collection, thereby ensuring methodological triangulation (Denzin, 1970) and researcher triangulation (Thurmond, 2001). In the qualitative phase, data were analyzed descriptively and supported by direct quotations (LeCompte and Goetz, 1982). Communication with multiple interviewers and participant verification enhanced the reliability of findings and confirmed the accuracy of interview responses (Başkale, 2016). To ensure the reliability of the scale, the Cronbach’s Alpha value was calculated. Following the necessary validity and reliability studies, the alpha value of the scale was found to be 0.732. This value is consistent with previous validation studies reporting satisfactory internal consistency. The researchers’ role was explained in the procedures section, and data sources were identified (LeCompte and Goetz, 1982). Written and digital data were systematically archived and kept accessible throughout the study (Guba and Lincoln, 1982). These measures strengthened the reliability, validity, and reproducibility of the qualitative data.

## 3 Findings

### 3.1 Findings related to pre-test scores

To conduct statistical analyses of participants’ pre-test scores, the data must be normally distributed, and the variances must be homogeneous (Field, 2013). Based on the hypothesis tests, the pre-test scores of both groups were normally distributed (*p*-values > 0.05). According to the Levene test results, the pre-test score variances of students in both groups were homogeneous (*p*-value > 0.05). After the necessary assumptions were met, an Independent Groups T-Test was conducted to determine whether there was a significant difference between the pre-test scores of the two groups. The findings are presented in Table 2.

**Table 2:** Results of the pre-test scores of the experimental and control group students on.

Measure	Experiment		Control		*t* (40)	*p*
	M	SD	M	SD		
Pre-test scores of groups	2.64	0.24	2.71	0.18	−0.981	0.333

Upon examination, there is no statistically significant difference between the pre-test score averages of the experimental group students and the control group students. This finding indicates that students’ Critical Thinking Skills Scale levels were similar before the application.

### 3.2 Findings regarding pre-test and post-test scores

The normality of the frequency distributions of the difference scores between the pre-test and post-test measurements for students in the experimental group was tested, and the distribution of the scores was found to be normal (*p*-value > 0.05). After the necessary assumptions were met, a Paired Samples T-Test was conducted to determine whether there was a significant difference between the pre-test and post-test scores of the experimental group students. The results are shown in Table 3.

**Table 3:** Results of the pre-test and post-test scores of the experimental group students on critical thinking skills scale.

Measure	Pre-test		Post-test		*t* (20)	*p*
	M	SD	M	SD		
Experimental group	2.64	0.24	3.46	0.15	−14.674	0.000

When the table is examined, it is determined that there is a statistically significant difference between the arithmetic mean of the pre-test scores and the arithmetic mean of the post-test scores of the experimental group students. The effect size value obtained is *ƞ*^2^ = 0.84. Based on this finding, it can be said that events supported by advertising films had a significant effect on the Critical Thinking Skills Scale development of students in the experimental group. Subsequently, the pre-test and post-test scores of the students in the control group were compared.

It was determined that the data followed a normal distribution, and a Paired Samples T-Test was conducted to determine whether there was a significant difference between the pre-test and post-test scores of the control group students. The findings are presented in Table 4.

**Table 4:** Results of the control group students’ critical thinking skills scale pre-test and post-test scores.

Measure	Pre-test		Post-test		*t* (20)	*p*
	M	SD	M	SD		
Control group	2.71	0.18	2.74	0.20	−1.579	0.130

When the table is examined, it is determined that there is no statistically significant difference between the arithmetic mean of the pre-test scores and the arithmetic mean of the post-test scores of the control group students.

Finally, an Independent Groups T-Test was performed to compare the final test scores of both groups. The normality test conducted previously indicated that the distributions of the final test scores for both groups were normal (*p*-value > 0.05). Findings related to the final test scores are presented in Table 5.

**Table 5:** Results regarding the final critical thinking skills scale test scores of the experimental and control group students

Measure	Experiment		Control		*t* (40)	*p*
	M	SD	M	SD		
Final test scores for groups	3.46	0.15	2.74	0.20	13.020	0.000

When the table is examined, it is determined that there is a statistically significant difference between the arithmetic mean of the final test scores of the experimental group students and the arithmetic mean of the final test scores of the control group students. The arithmetic means of the experimental group students are greater than the arithmetic means of the control group students. The effect size obtained is *ƞ*^2^ = 0.80. The effect size (*η*^2^ = 0.80) indicates a very large effect according to commonly accepted benchmarks, suggesting that advertising film-based instruction had a strong educational impact on students’ critical thinking development.

### 3.3 Findings regarding final test and follow-up test scores

First, the normality of the frequency distributions of the difference scores between pre-test and post-test measurements for the experimental group was confirmed (*p*-value> 0.05). Subsequently, a Paired Samples T-Test was conducted to determine whether there was a significant difference between the final test and follow-up test scores of the experimental group students. The findings are presented in Table 6.

**Table 6:** Results regarding the final test and follow-up test scores of the experimental group students.

Measure	Post-test		Follow-up test		*t* (20)	*p*
	M	SD	M	SD		
Experimental group	3.46	0.15	3.48	0.16	−1.338	0.196

When the table is examined, it is determined that there is no statistically significant difference between the arithmetic mean of the final test scores of the experimental group students and the arithmetic mean of the follow-up test scores. According to the findings, there was no significant change in the Critical Thinking Skills Scale levels of the students in the experimental group.

### 3.4 Findings obtained through observation

**Figure 1:**
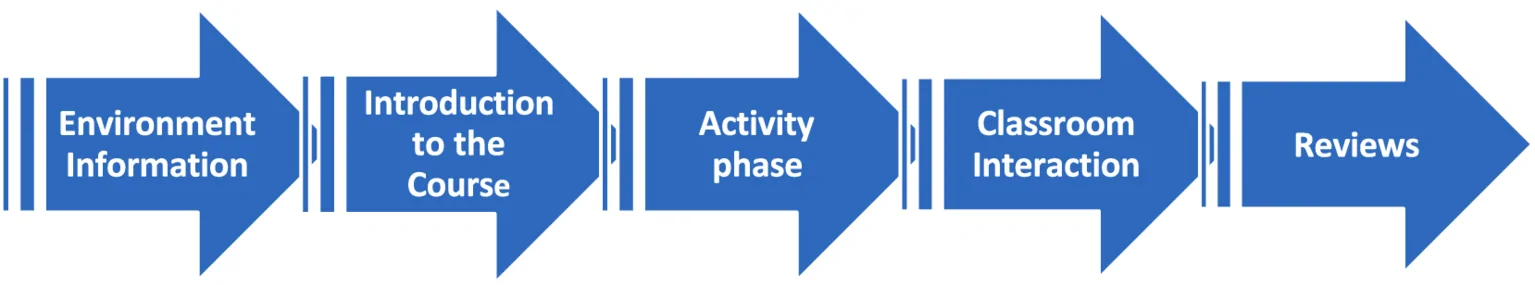
Themes created for the analysis of observation forms.

This section presents findings from semi-structured observations to answer the third research question. Observations were conducted by observing the students in the experimental group in weeks 2, 4, 6, and 8. Classroom observations were conducted by four trained observers who independently recorded student behaviors during instructional activities. The themes presented in Figure 1 have been created to clearly and concisely present the findings to the reader. Observers are coded as G1 and G2, and their opinions are presented accordingly.

#### 3.4.1 Environment information

The environmental knowledge theme comprises two subcategories: physical and social environments. The observation form enabled observers to monitor the physical setting of advertising film-based activities, focusing on aspects such as temperature, lighting, equipment, seating arrangements, and student numbers. G1 emphasized temperature and lighting; G2 mentioned lighting and classroom size; G3 focused on the interaction between desks and the interactive whiteboard; and G4 noted the space relative to the number of students. G1 stated, “The physical environment is suitable in terms of temperature and lighting,” while G4 said, “The classroom is large enough considering the number of students. Students are seated comfortably.” These views suggest the physical environment supported student participation.

In the social environment category, relationships between students and the teacher stood out. G1 observed that students respected each other’s rights and listened to peers. G2 highlighted the importance of positive communication and maintaining eye contact between students and the teacher. G3 noted that most students were eager to share ideas, and G4 stated that the teacher was sincere and cheerful, with students generally following classroom rules.

#### 3.4.2 Introduction to the course

Within this theme, the relevant codes were examined, and categories were created to attract attention, arouse curiosity, and activate prior knowledge. Observers first emphasized the materials the teacher used at the beginning of the lesson, noting that they attracted students’ attention. G1 said, “The teacher entered the classroom with a bowl full of candy and an interesting picture, which made students who were distracted suddenly focus on him.” G2 stated that the images the teacher posted on the board at the beginning of the lesson sparked curiosity, and that students began examining them with interest. G3 said, “The teacher entered the classroom holding a tree seedling and a picture of a desert. Suddenly, the students looked at him, trying to understand what he was holding.” According to G3, students were motivated to attend class because they believed they would engage in different activities. G4 stated that the teacher began the lesson with a question that activated prior knowledge, followed by information on the lesson objectives, thereby ensuring more active student participation.

#### 3.4.3 Activity phase

The activity phase is divided into two categories: ensuring participation and developing critical thinking skills. Observers first noted that students attentively watched the advertising films, asked questions, tried to understand the topics, and actively engaged in the lesson. G1 stated, “Activities should attract students’ attention, stimulate their imagination, and arouse curiosity to facilitate understanding.” Similarly, G4 observed, “Students participated voluntarily and seemed eager to express their opinions.”

The second category focused on how activities contributed to the development of critical thinking. G2 noted, “Students analyzed and commented on the topics through the videos and subsequent activities.” G3 highlighted that students shared their views confidently during class. Other observers agreed that the activities encouraged skills such as analysis, synthesis, and evaluation, supporting the development of critical thinking. G2 emphasized, “The activities supported students’ reasoning skills, such as analysis and evaluation.” These observations suggest that the activities may have promoted both participation and critical thinking.

#### 3.4.4 Classroom interaction

Based on observations of classroom interaction, two categories emerged: student-teacher and student–student interaction. The teacher was observed to be sincere, listening carefully to students’ questions and offering clear explanations. G3 noted that students felt comfortable asking questions without hesitation. G1 stated, “During activities, students listen carefully to the teacher and take notes. When an incorrect answer is given, the teacher provides feedback or makes corrections.” G2 observed, “The teacher entered with a sincere expression and greeted students with a smile. The students responded similarly,” reflecting positive student-teacher interaction. G4 highlighted that students asked each other questions during activities and listened respectfully. Overall, the findings suggested that mutual, respectful, and supportive interaction may have contributed to participation.

#### 3.4.5 Reviews

The assessment theme consists of the categories *student products* and *assessment/perception skills*. Observers who paid attention to the tangible products students created during the activities noted that the products were suitable for their intended purpose and successful. G1 stated, “Students wrote essays describing their dreams for the future and their desired professions using writing strategies.” G2 stated that pictures appropriate to the video content watched in class were drawn and posted on the board. G3 indicated that students were able to perform activities targeting higher-level mental skills, such as predicting the title and outcome of a story. G4 indicates that students were able to identify the topic of the video watched during the activity, state the main idea, evaluate it, and write original texts appropriate to the main idea. The observation data suggest that the activities designed to develop critical thinking skills may have achieved their intended purpose.

### 3.5 Findings obtained through interviews

**Figure 2:**
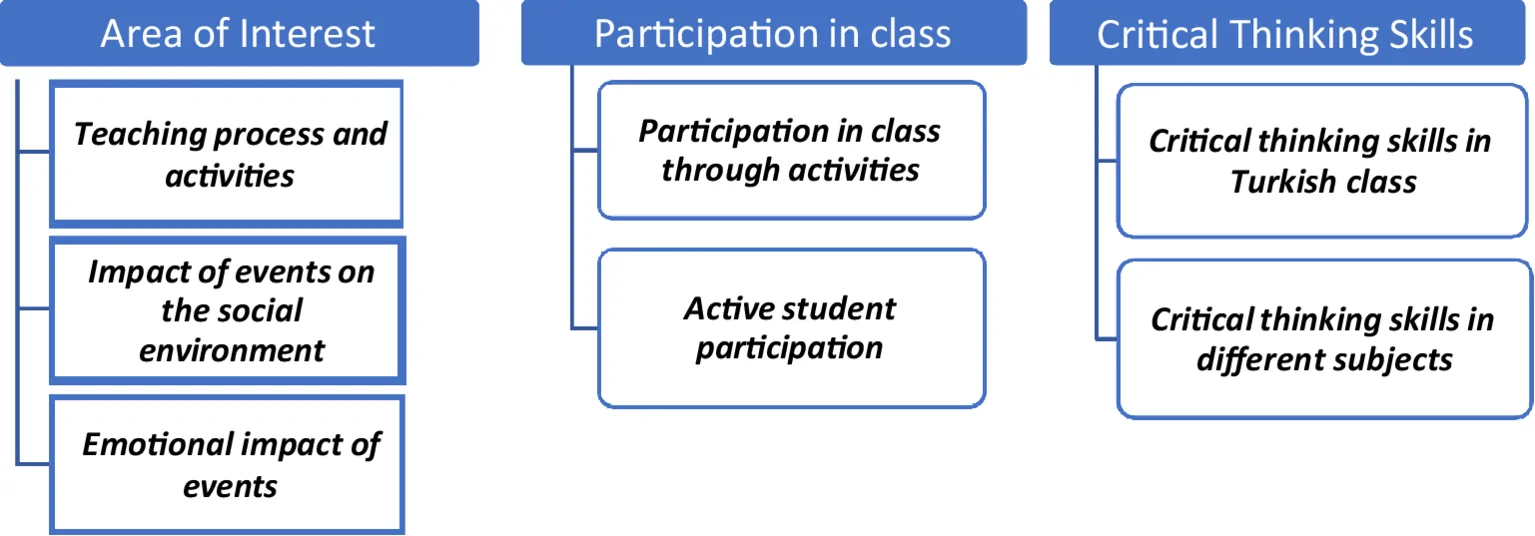
Themes and categories created for the analysis of interview forms.

This section presents findings from student interviews in response to the fourth research question. The themes that emerged from the interviews were centered around the concepts of *area of interest, class participation*, and *critical thinking skills*. The categories related to these themes, along with the number of codes for each, are shown in Figure 2.

#### 3.5.1 Area of interest

##### 3.5.1.1 Teaching process and activities

Codes related to the category of “teaching process and activities” have been determined as “making the learning process understandable, enriching the lesson process with activities, structuring the lesson in an interesting and motivating way, drawing students’ attention to the content of the videos, and going beyond the existing process with the activities used.” One of the students (Ö3) said, “The activities we did in class were exciting, and I started listening to the lesson with curiosity and attention.” Another student (Ö2) said, “We taught the lesson using a different method, and all my friends observed the videos in class,” expressing that the activities were engaging and motivating.

##### 3.5.1.2 Impact of events on the social environment

Codes related to the category “Impact of activities on the social environment” have been determined as “activities that encourage students to share their thoughts and activities that develop students’ social skills.” Student code Ö2 said, “I was hesitant to express my thoughts, but after this class, I started to gain confidence in myself, and I began to enjoy it.” Ö1 expressed his thoughts by saying, “We discuss the events in the movies we watch with my friends. We listen to each other.”

##### 3.5.1.3 Emotional impact of events

Codes belonging to the category of “emotional impact of activities” are listed as “activities that develop students’ critical thinking skills, activities that motivate students to participate in class, and activities that stimulate students’ imagination.” One of the students (S5) said, “The films I watched had a profound effect on me; some made me laugh a lot, while others moved me.” Some videos made us think and helped us gain awareness.” Therefore, it can be said that the activities had an emotional impact on the students.

#### 3.5.2 Participation in class

##### 3.5.2.1 Participation in class through activities

This category includes codes such as facilitating understanding of the lesson through activities, films used in activities, students’ desire to analyze advertising films, the motivational effect of activities, and fun lessons. During the interview, one student (Ö6) said, “The activities and films made the class more interesting; I looked forward to coming to class every week because I wondered what we would do.” Another student (Ö9) said, “I did not notice how time passed during the activities because I was having so much fun. Like the rest of the class, I wanted to participate in the class actively.” Ö11 stated that events featuring commercials were interesting. As a result, the findings suggest that the activities may have encouraged student participation in class and may have supported the development of critical thinking skills.

##### 3.5.2.2 Active student participation

Codes related to active student participation include asking questions in class, participating in group work, expressing opinions in discussions, attempting to solve problems, and managing one’s learning. During the interview, one student (Ö12) said, “We asked each other questions during the activities; although I was shy about expressing my ideas at first, I gained confidence over time.” Another student (Ö8) said, “The group discussions were quite interactive and exciting; all group members spoke up and shared their opinions. The fact that other participants listened to me attentively increased my interest and motivation in the course. Ö7 stated that his self-confidence increased as he successfully solved problems during the activities. Based on student feedback, the activities increased students’ active participation in class, supported their self-directed learning, and improved their problem-solving and evaluation skills.

#### 3.5.3 Critical thinking skills

##### 3.5.3.1 Critical thinking skills in Turkish class

This category includes codes related to the development of language skills (listening, reading, speaking, and writing), improved comprehension of what is read, enhanced expression skills, and increased academic success in Turkish language classes. The students interviewed stated that the activities helped them express themselves more effectively and accurately. Ö6 “After participating in the activities, I noticed that my speaking skills had improved and that I was able to form sentences more correctly. I think I am better at written expression.” Ö11, one of the students interviewed, said, “I realized that I have developed the ability to make inferences about the texts I read. This development was also reflected positively in my Turkish exam results, and I received a high grade.” Thus, he emphasized the impact of the activities on academic achievement. Ö15, “When I listened carefully to my friends’ thoughts, I saw that they could also be right. This situation made me realize that I had not been using my listening skills effectively in the past. Ö8 expressed his thoughts as follows: “I am now more successful in Turkish class, so my self-confidence has increased.” Student feedback indicates that the activities improved their reading comprehension and interpretation skills, as well as their language skills, thereby enhancing their academic performance.

##### 3.5.3.2 Critical thinking skills in different subjects

This category encompasses analytical, creative, questioning, and critical thinking, as well as finding alternative solutions and other essential skills for success across various *subjects*. The students interviewed stated that their academic performance improved in school exams and mock exams for different subjects after participating in the activities implemented in Turkish language classes. Ö2 said, “After the activities, I saw that I could analyze a text in detail.” Ö14 “We tried to develop different solutions to the questions posed by our teacher and focused on generating original ideas. I believe that such activities have a positive impact on my academic performance, particularly in Social Studies and other subjects. I can now see that I can give more effective and comprehensive answers to questions in different subjects.” Ö8 said, “I developed my critical thinking skills through activities; now I try not to accept information I hear without questioning it and researching its accuracy.” In addition, I have gained the courage to defend my own opinions, even though they are often considered different. I strive to maintain this attitude in all my classes.” Ö13 stated that he could think creatively, especially in mathematics and science classes, and that his performance had improved.

Although most participants reported positive learning experiences, the analysis also highlighted varying levels of engagement among students, indicating differences in individual responses to multimodal learning activities. When student opinions are evaluated overall, it is understood that activities supported by advertising films improve students’ comprehension and interpretation skills and increase interest, participation, and academic achievement in other courses as well. The qualitative findings revealed interconnected themes of student engagement, cognitive awareness, and critical interpretation, which collectively reflected the development of higher-order thinking skills.

## 4 Discussion

### 4.1 Findings related to research question 1 and research question 2

The findings of this study demonstrated a statistically significant improvement in the critical thinking skills of the experimental group. Additionally, significant differences were found between the experimental and control groups in favor of the experimental group. These results extend previous research by providing experimental evidence not only on the development but also on the retention of critical thinking skills through advertising film-based instruction in middle school contexts. In contrast to traditional instruction, the integration of multimodal materials appears to create richer learning environments that support higher-order thinking.

From a constructivist perspective, learning occurs through active meaning-making processes, and multimedia-supported materials encourage students to question, evaluate, and reconstruct knowledge. In this context, advertising films, with their multimodal structures combining visual, verbal, symbolic, and narrative elements, promote cognitive engagement by requiring learners to interpret implicit meanings and analyze persuasive messages. This process is further supported by multimodal learning theory and critical literacy perspectives, which emphasize the integration of multiple representational modes and the questioning of ideological meanings in media texts.

Consistent with previous studies (e.g., Farías and Seguel, 2014; Checa-Romero, 2016; Zalani et al., 2024), advertising films and similar multimodal texts foster analytical and interpretive skills by exposing students to layered meanings and persuasive strategies. The linguistic and rhetorical features of advertising films further encourage critical language awareness and message evaluation (Koonnala and Chaiwong, 2023; Stanley and Lawson, 2020).

### 4.2 Findings related to research question 3

Qualitative findings based on classroom observations revealed increased student engagement, motivation, and active participation during the advertising film-based activities. Importantly, while most responses were positive, variation in engagement levels indicated individual differences in response to multimodal learning environments. These findings demonstrate how observational data help explain classroom processes underlying the quantitative improvements.

### 4.3 Findings related to research question 4

Qualitative interview data showed that students generally expressed positive opinions about the activity process. They reported that advertising films made lessons more interesting, engaging, and understandable. These findings support the view that multimodal instructional environments can enhance learner motivation and participation, while also highlighting the role of individual differences in learners’ perceptions.

### 4.4 Integrated interpretation of findings

These results are consistent with contemporary perspectives on multimodal literacy and literature education, which conceptualize literature as a dynamic, multimodal structure (Luukka, 2021; Calafato and Hunstadbråten, 2024). Such perspectives support the integration of media-based texts into language education, as literary engagement inherently involves interpretation, symbolic reasoning, and evaluation of perspectives (Tsang et al., 2023; Bobkina et al., 2021).

Although some studies highlight potential limitations of digital media such as cognitive overload or distraction (Tobias and Fletcher, 2011), no such effects were observed in this study due to the pedagogically structured design of the intervention. Moreover, the retention test results indicate that the effects of the intervention were sustained over time, supporting previous findings on the durability of reflective thinking (Gürbüztürk and Ünal, 2022).

Finally, this study contributes to the literature by employing a mixed-methods design that integrates quantitative and qualitative data. This approach provides a more comprehensive understanding of how advertising films influence critical thinking development by combining statistical evidence with learners’ experiential perspectives.

## 5 Conclusion

This study demonstrates that advertising films as multimodal learning materials significantly enhance students’ critical thinking skills while also supporting active engagement, interpretation, and reflection in language learning contexts. The findings indicate that integrating advertising films into instructional processes transforms learning from passive reception into active meaning-making and critical analysis.

The results are consistent with previous research emphasizing the role of multimodal and media-based texts in promoting higher-order thinking skills such as analysis, interpretation, and evaluation. However, this study extends the literature by providing experimental evidence on both the development and retention of critical thinking skills through advertising film-based instruction in a middle school context, thereby contributing new insights into the sustainability of cognitive gains.

From a practical perspective, advertising films can be integrated into language education through structured activities that encourage students to analyse persuasive strategies, interpret symbolic meanings, and evaluate multiple perspectives. Such practices may be incorporated into curriculum design, classroom instruction, and teacher training programs to support multimodal literacy and critical thinking development.

This study has several limitations. The sample consisted of 44 seventh-grade students from a single school in eastern Turkey with similar socio-economic backgrounds, which may limit generalizability. The quasi-experimental design and short follow-up period further constrain the interpretation of long-term effects. Additionally, the study focused on a single subject area and a specific type of multimodal content. Future research should include larger and more diverse samples, different educational contexts, and longer follow-up periods to strengthen the generalizability of findings.

Overall, the study highlights the educational potential of advertising films as multimodal texts that bridge media literacy and critical thinking development in language education.

## Data Availability

The datasets presented in this study can be found in online repositories. The names of the repository/repositories and accession number(s) can be found below: https://drive.google.com/drive/folders/1fe1w621rkl-5-hhA9C7t5buQAqkbScFx?usp=drive_link.
